# Metallic and Ceramic Thin Film Thermocouples for Gas Turbine Engines

**DOI:** 10.3390/s131115324

**Published:** 2013-11-08

**Authors:** Ian M. Tougas, Matin Amani, Otto J. Gregory

**Affiliations:** Department of Chemical Engineering, University of Rhode Island, 16 Greenhouse Road, Crawford Hall Room 205, Kingston, RI 02881, USA; E-Mails: itougas007@gmail.com (I.M.T.); matin.amani@gmail.com (M.A.)

**Keywords:** thin film, gas turbine engine, thermocouple, platinum, palladium, indium tin oxide, sputtering

## Abstract

Temperatures of hot section components in today's gas turbine engines reach as high as 1,500 °C, making *in situ* monitoring of the severe temperature gradients within the engine rather difficult. Therefore, there is a need to develop instrumentation (*i.e.*, thermocouples and strain gauges) for these turbine engines that can survive these harsh environments. Refractory metal and ceramic thin film thermocouples are well suited for this task since they have excellent chemical and electrical stability at high temperatures in oxidizing atmospheres, they are compatible with thermal barrier coatings commonly employed in today's engines, they have greater sensitivity than conventional wire thermocouples, and they are non-invasive to combustion aerodynamics in the engine. Thin film thermocouples based on platinum:palladium and indium oxynitride:indium tin oxynitride as well as their oxide counterparts have been developed for this purpose and have proven to be more stable than conventional type-S and type-K thin film thermocouples. The metallic and ceramic thin film thermocouples described within this paper exhibited remarkable stability and drift rates similar to bulk (wire) thermocouples.

## 1. Introduction

The next generation of gas turbine engines will employ advanced materials, which are designed to handle the harsh environments inside the hot section of these engines [1]. With advances in engine materials comes the need for developing new instrumentation which can handle the higher temperatures, pressures and gas flow to monitor the operating conditions inside the engine during operation. Engine designers must gain an understanding of the operating conditions inside the engine and perform diagnostics in order to ensure it is operating safely and according to the final design parameters. Specifically, improvements such as lighter engine components with superior thermomechanical properties, advanced thermal barrier coatings (TBC) for turbine blades, and higher operating temperatures to improve overall combustion efficiency and reduce harmful emissions are placing greater demands on the instrumentation itself. Therefore, the design and implementation of sensors to monitor operating conditions and perform diagnostics in these engines during testing is becoming an increasingly difficult task.

Many issues arise when attempting to integrate sensors inside gas turbine engines, considering they operate under severe conditions where supersonic nozzle velocities, rotational forces exceeding fifty thousand g, and gas temperatures reaching as high as 1,500 °C in the hot section are present. Conventional wire sensors cannot reliably withstand these conditions due to their relatively large thermal masses and surface profiles that extend beyond the boundary layer of rotating parts, which can affect combustion gas flow patterns and induce mechanical interference with the vibrational modes of rotating parts. As blades and other components get thinner in cross section, efforts have focused on using thin film sensors to replace wire sensors. Thin film sensors do not interfere with gas flow patterns because they have thicknesses on the order of micrometers, well below the boundary layer thickness of instrumented engine component surfaces [2,3]. And since the masses of the sensors are on the order of micrograms, they do not affect the vibrational modes of rotating parts and are not affected by the rotational forces inside the engine [3]. Thin film sensors have much faster signal response times due to lower thermal mass relative to wire sensors [4]. Thin films sensors are directly deposited onto the surface of thermal barrier coatings of stationary and rotating components without the need for high temperature adhesives. As a result, more accurate surface measurements are possible [1,2,5].

Oxidation in the gas turbine engine environment can cause instability and drift in metallic thin film thermocouples due to the significantly reduced diffusional distances relative to wire thermocouples. For example, in type-S thin film (platinum: 90%-platinum-10% rhodium) thermocouples, selective oxidation of rhodium in the platinum rhodium thermocouple leg forms volatile rhodium oxides at temperatures between 600–800 °C, and can cause degradation of these temperature sensors [6]. The selective oxidation process at high temperature is progressive and time dependent, causing drift in the thermoelectric output by as much as 13.8 °C/h as the platinum-rhodium thermocouple legs undergo both microstructural and compositional changes [2]. Furthermore, the same effect can be seen in type-K (alumel:chromel) thin film thermocouples at substantially lower temperatures. Therefore, more stable metallic thin film thermocouple combinations must be considered, which do not experience detrimental microstructural changes and selective oxidation issues at high temperatures.

Wire thermocouples based on platinum and palladium have been investigated to some extent [7–11], however, investigations of thin film Pt:Pd thermocouples have been somewhat limited. These materials, being elemental metals, avoid many of the issues exhibited by alloy thermo-element materials in high temperature environments, such as selective oxidation seen in type-S thin film thermocouples. The use of thin film Pt:Pd thermocouples on oxidized silicon wafers for radiometric temperature measurements has been considered by Kreider *et al.* up to 1,050 °C [12,13]. However, due to dewetting issues, these thermocouples could only reliably measure temperatures up to 850 °C and required the use of a titanium bond coat between the silicon wafer and thermocouple elements [12,13].

Additionally, research has focused on ceramic thin film thermocouples as an alternative to metallic thin film thermocouples. Ceramics have high melting temperatures and chemical and electrical stability at high temperatures in oxidizing atmospheres. They also have excellent thermal expansion coefficient compatibility with ceramic thermal barrier coatings applied to the surface of engine components. TiC-TaC thin film thermocouples, while showing promising results up to 1,080 °C, are limited to inert atmospheres or vacuum conditions due to thermo-element degradation in the presence of oxygen at high temperature [14]. Silicides have also been investigated for thin film thermocouples [6]. MoSi_2_ and TiSi_2_ proved to be stable in air to 1,200 °C due to a protective silicon oxide layer which grew over the surface of the thermo-elements during thermal cycling. However, this consumes the silicon in the film, changing the chemical composition of the thermo-element, which can lead to drift. To compensate for this loss, a layer of silicon must be deposited beneath the thermo-element. Moreover, silicide- and carbide-based thin film thermocouples have low outputs compared to type-S thin film thermocouples.

Oxides have proven to be more stable as thermo-elements in harsh environment since they contain all the oxygen their structure can accommodate. Indium oxide (In_2_O_3_) and indium tin oxide (ITO) are wide band gap semiconductors commonly used in transparent conductor applications, and when In_2_O_3_ contains low concentrations of extrinsic dopants (*i.e.*, tin), oxygen vacancies defects are often the primary charge carrier. This defect is defined according to Equation (1):

(1) $$O_{0}^{x}\to \frac{1}{2}O_{2}+V_{\ddot{O}}+2e^{-1}$$

where ${\text{V}}_{\text{O}}^{\cdot \cdot }$ are doubly charged oxygen vacancies. In_2_O_3_ contains unoccupied oxygen interstitials preserved in its cubic bixbyite crystal structure [15], so upon thermally cycling at high temperature, penetration of oxygen into the film which compensates defect oxygen vacancy sites cannot chemically accommodate these built-in lattice interstitial sites. Therefore, thermally cycled In_2_O_3_ thin films remain conductive at room temperature with resistivities as low as 0.02 Ω·cm despite partial compensation of oxygen vacancy defect charge carriers. The temperature dependence of the electrical resistivity of ITO thin films after heat treatment in air has a distinct transition in activation energy corresponding to the excitation of all dopant species to the conduction band, which occurs between 600 °C and 700 °C [16]. This phenomenon makes oxides more sensitive thermocouples materials than metals at temperature above 1,000 °C because metals do not see the same level or abrupt change in conductivity with temperature as semiconducting oxides. The same oxygen vacancy induced charge carrier is also observed in zinc oxide (ZnO), however, it does not have the same benefit of a lattice-preserved interstitial oxygen vacancy site observed in indium oxide, and therefore exhibits increased drift and instability during thermal cycling. ITO with up to 10 wt% tin forms a solid solution in In_2_O_3_, which retains the bixbyite structure, and acts as an n-type dopant to increase the carrier concentration. Therefore, the oxygen vacancy preservation also occurs in ITO up to a 90%/10% indium oxide/tin oxide composition.

ITO has improved electrical conductivity over In_2_O_3_ because the inclusion of two tin and one oxygen atoms forms a neutral charge carrier site, in addition to self-doping caused by intrinsic defects. Since impurities tend to collect at grain boundaries, the electrical conduction mechanisms in these materials are dictated by the grain boundary area per unit volume. During thermal cycling, these materials undergo sintering which results in changes to the grain boundary area and therefore charge carrier concentration, eventually leading to drift [17]. As previously demonstrated, an all-ceramic thin film thermocouples based on In_2_O_3_ and ITO exhibited higher sensitivites than metallic thin film thermocouples with an order of magnitude greater Seebeck coefficient 170 μV/°C at 1,250 °C [18]. However, this same thin film thermocouple had a drift rate of 3.76 °C/h which is an order of magnitude higher than a commercially available metal wire thermocouple.

In this manuscript we review several developments which have recently been demonstrated to improve the performance of thin film thermocouples. Specifically we focus on the development of thin film Pt:Pd and indium oxynitride:indium tin oxynitride (InON:ITON) thin film thermocouples along with their oxide counterparts, which have been deposited onto ceramic substrates via radio frequency sputtering. The thermoelectric properties of Pt:Pd thin film thermocouples were compared to type-S and type-K to determine the benefits of using single element materials rather than alloys for thin film thermocouples in harsh oxidizing environments. The stability of In_2_O_3_:ITO thin film thermocouples was improved via nitrogen plasma processing. Due to the sluggish sintering kinetics of nitrides *versus* oxides, nitrogen plasma processing was used to fabricate InON:ITON thin film thermocouples to improve their stability over the oxide counterparts. The thermoelectric output and drift were measured at temperatures up to 1,100 °C for Pt:Pd and 1,400 °C for InON:ITON thin film. Both exhibited remarkable stability at high temperature in an oxidizing environment. The electrical resistivity and densification as a function of time at temperature of the ceramic thin film thermocouples was measured. Auger electron spectroscopy (AES) was used to characterize the chemical composition of the Pt:Pd metallurgical junctions as a function of depth and to monitor the extent of oxidation. The microstructure of the thin film thermocouples were examined using scanning electron microscopy (SEM).

## 2. Experimental Section

**Figure 1.**
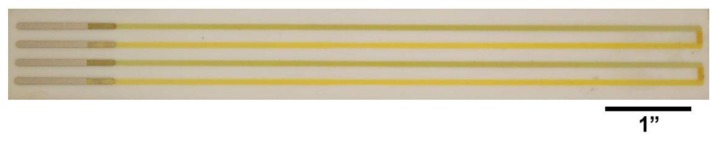
Two In_2_O_3_:ITO 90/10 thin film thermocouples fabricated by radio frequency sputtering onto a ceramic substrate.

All thin film thermocouples were deposited onto either alumina or mullite ceramic substrates (CoorsTek Inc., Golden, CO, USA) which measured 190 mm × 25 mm × 2 mm. Each substrate was cleaned with acetone, methanol, and deionized water. A dry film negative photoresist (MX 5050™, DuPont™, Wilmington, DE, USA) was soft-baked to the surface of the substrates and bond pad patterns were transferred to the photoresist using a 350 nm wavelength ultraviolet light Optical Associates, Inc. aligner (San Jose, CA, USA) to expose the resist. All optimized sputtering parameters for the various films used in this study are shown in Table 1 and all films were deposited either in a model 8667 or 822 Materials Research Corporation (now owned by a subsidiary of Sony Corporation of North America, Praxair, Danbury, CT, USA) sputtering system. An initial photolithography step was performed prior to depositing the thermocouple elements to pattern platinum bond pads. A second photolithography step transferred a thin film thermocouple leg pattern to the resist using the same ultraviolet light exposure. Windows of the desired patterns were developed in the film after exposure and the first thin film thermocouple leg was sputtered. The second photolithographic step was repeated to fabricate the second thin film thermocouple leg. Films were deposited in pure argon except for the oxynitride materials which were sputtered in a combination of argon and nitrogen, where the total gas pressure was 1.2 or 1.33 Pa for metals and ceramics, respectively. A background pressure of 2.7 × 10^−4^ Pa was achieved in the sputtering chamber prior to sputtering, the stage (work piece) was maintained at temperatures below 100 °C using water-cooling, and the targets were pre-sputtered onto shutters for 10 min to remove surface contamination and release adsorbed moisture. Metal thin film thermocouples were anywhere from 1–5 μm thick and ceramic thin film thermocouples were 10–12 μm thick, depending on the material. Fully fabricated thin film thermocouples on a ceramic substrate are shown in Figure 1.

All thin films were annealed in nitrogen for 5 h at 500 °C to remove point defects, including trapped argon, and densify the films. A second anneal was performed in air for 2 h at 1,200 °C to ceramic thin film thermocouples only. This was intended to expose the entire thermocouple to a temperature over 1,000 °C prior to testing to prevent the hot and cold junction from sintering and oxidizing unevenly when applying the thermal gradient induced during testing. Uneven heating creates inhomogeneities in the microstructure and extent of sintering, which leads to drift and eventually device failure. The annealing step prevents this from occurring and stabilizes each thin film thermocouples for longer duration use.

**Figure 2.**
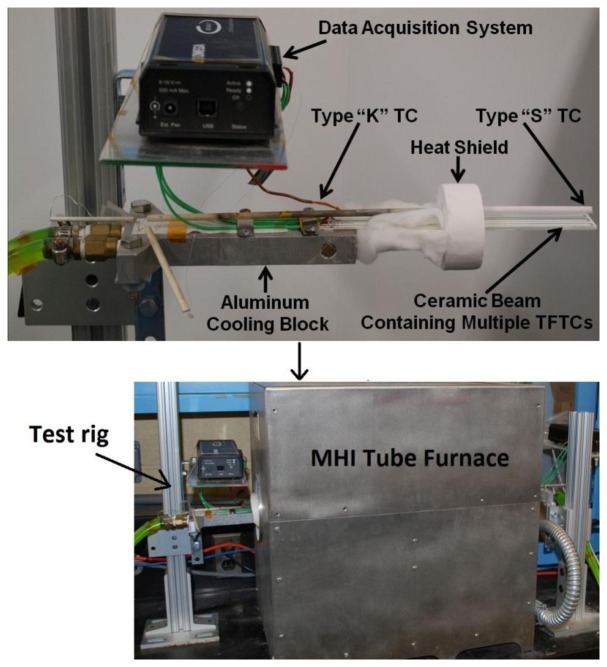
Custom tube furnace testing rig for thin film thermocouples deposited on ceramic beams, which applied a horizontal temperature difference along the length of the thermocouple measured with type-K and type-S wire thermocouples. This setup simulated the severe temperature gradients on the surface of components inside gas turbine engine hot sections.

The thermoelectric output of each thin film thermocouple was measured as a function of time and temperature by placing the hot junction in the hot zone of a tube furnace and attaching the cold junction to an aluminum chill block outside the furnace. In this way, a temperature difference was applied along the length of the beam and the cold junction was maintained at or below 100 °C using chilled water or ethylene glycol. Since the temperature of the cold junction could not be maintained at a constant value due to cooling limitations, a wire type-K thermocouple was used to measure the temperature on the cold side of the ceramic substrate. The test apparatus used for all experiments is shown in Figure 2. The thermal cycling protocol is as follows: heat from room temperature at 4 °C/min to desired peak temperature, hold for 10 min, cool from peak temperature to 250 °C at 4 °C/min and hold for 6 min, repeat first cycle from 250 °C to peak temperature and back at the same rate, heat to peak temperature and hold for 10 h, cool to 250 °C and hold for 6 min, repeat second cycle, and cool to room temperature. Pt:Pd thin film thermocouples were thermally cycled in air to 900 °C for several cycles to determine the thermoelectric voltage and drift rate over a 10 h period. In_2_O_3_:ITO-based thermocouples were thermally cycled to 1,200 °C for several cycles and their long-term stability was obtained by holding at a temperature difference of 1,200 °C for 200 h. The temperature difference was continuously monitored using type-K (cold junction) and type-S (hot junction) wire thermocouples and copper extension wires were attached to the platinum bond pads of the Pt:Pd thin film thermocouples using a silver paste to acquire the voltage signals. All copper wires and wire thermocouples were connected to an Personal Daq 54 USB data acquisition system (Measurement Computing, Norton, MA, USA) with *PDaq View Plus^©^* software recording the temperature and voltage signals. The Seebeck coefficient and drift rates of the thin film thermocouples were determined as a function of hot/cold junction temperature and time, respectively. The Seebeck coefficient defined in this study is given by Equation (2):

(2) $$S=\frac{-\Delta V}{\Delta T}=-\left(\frac{\Delta V_{a}-\Delta V_{b}}{T_{h}-T_{c}}\right)$$

where *S* is the Seebeck coefficient given in μV/°C, *ΔV* is the voltage potential difference between the two thermocouple materials (*ΔV_a_*− *ΔV_b_*), and *ΔT* is the temperature difference between the hot junction (*T_h_*) and the cold junction (*T_c_*). Additionally, thermoelectric data for type-K and type-S thin film thermocouples prepared using the same photolithographic and vacuum deposition methods is given for comparison to the Pt:Pd thin film thermocouples.

To better understand the electrical properties of the semiconducting oxide- and oxynitride-based thermocouples, the resistivity was determined as a function of temperature using the van der Pauw method as a function of temperature and is described by Equation (3):

(3) $$\sigma =\sigma_{0}\exp(-\Delta {\text{E}}_{\text{a}}/\text{kT})$$

where k is Boltzmann's constant, T is the absolute temperature, σ_0_ is a pre-exponential factor, σ is the electrical conductivity, and ΔE_a_ is the activation energy which is the energy gap between the conduction band edge and the Fermi level. The sintering and growth kinetics of In_2_O_3_ films sputtered in different argon, oxygen and nitrogen partial pressures deposited on highly polished alumina wafers was measured using a Dektak IIA (currently Bruker, Camarillo, CA, USA) surface profilometer to determine the change in thickness of films as a function of time at temperature. Auger electron spectroscopy (AES) depth profiles were used to determine the chemical composition of the metallurgical junction and identify any oxidation formed on the platinum and palladium thermocouple legs. AES was performed using a 5500 Multi-Technique Surface Analyzer (Perkin Elmer, Waltham, MA, USA). A background pressure of 6.0 × 10^−7^ Pa was established and a 1 × 1 mm area on the surface of each thin film thermocouple was sputter cleaned for 10 s prior to acquiring each depth profile. SEM was performed on the same films to analyze the microstructure after thermal cycling using a JSM-5900LV SEM (JEOL, Peabody, MA, USA) and a 20 kV accelerating voltage. SEM micrographs of oxide- and oxynitride-based thermocouple cross-sections and film surfaces were also taken using the same equipment and parameters. Additionally, the oxide and oxynitride films were deposited onto sapphire substrates and grazing incidence X-ray diffraction (GI-XRD) using a Cu_Kα_ radiation source was used to identify the phases present in the thin films.

## 3. Results and Discussion

### 3.1. Thermoelectric Measurements

#### 3.1.1. Type-K and Type-S Thin Film Thermocouples

**Figure 3.**
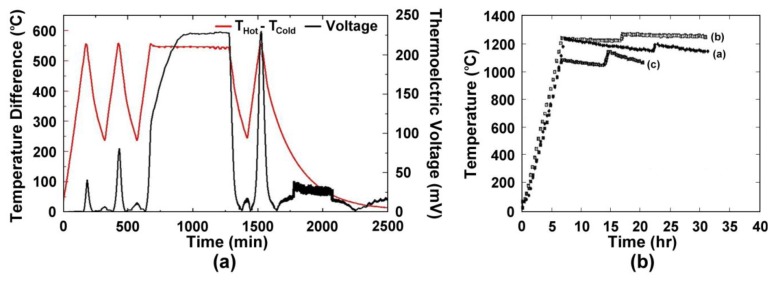
(**a**) Thermoelectric output of a type-K thin film thermocouple on alumina and (**b**) several type-S thin film thermocouples on silicon nitride from a previous study. The type-S data set were for (a) air ambient, 300 °C temperature difference, (b) nitrogen ambient, 300 °C temperature difference, and (c) air ambient, 600 °C temperature difference.

Wire thermocouples based on alumel:chromel (type-K) and platinum:platinum-10% rhodium (type-S) are commonly used for high temperature measurement in the 500–1,500 °C range. However, many other issues arise for thin films of these materials that reduce their performance. Figure 3 shows the time, temperature, thermoelectric response of type-K (a) and type-S (b) thin film thermocouples studied in previous work [2]. The type-K thin film thermocouple shown in Figure 3a was unstable even at low temperatures. Due to the much shorter diffusional length of oxygen through the film *versus* a wire (micrometers *versus* millimeters) and oxidation of the films during thermal cycling, drift was prominent and the device failed to perform stably over long periods of time. The increase in peak thermoelectric voltage is attributed to microstructural changes in the film, such as oxidation, as well as progressive dewetting of the film and evaporation. Figure 3b shows three different type-S thin film thermocouples, which were subjected to different thermal cycling ambient and temperature differences from a previous study [2]. The instability of these thermocouples made them unsuitable for use in harsh high temperature environments and therefore thin film thermocouples based on refractory metals and ceramics were investigated.

#### 3.1.2. Platinum: Palladium Thin Film Thermocouples

**Figure 4.**
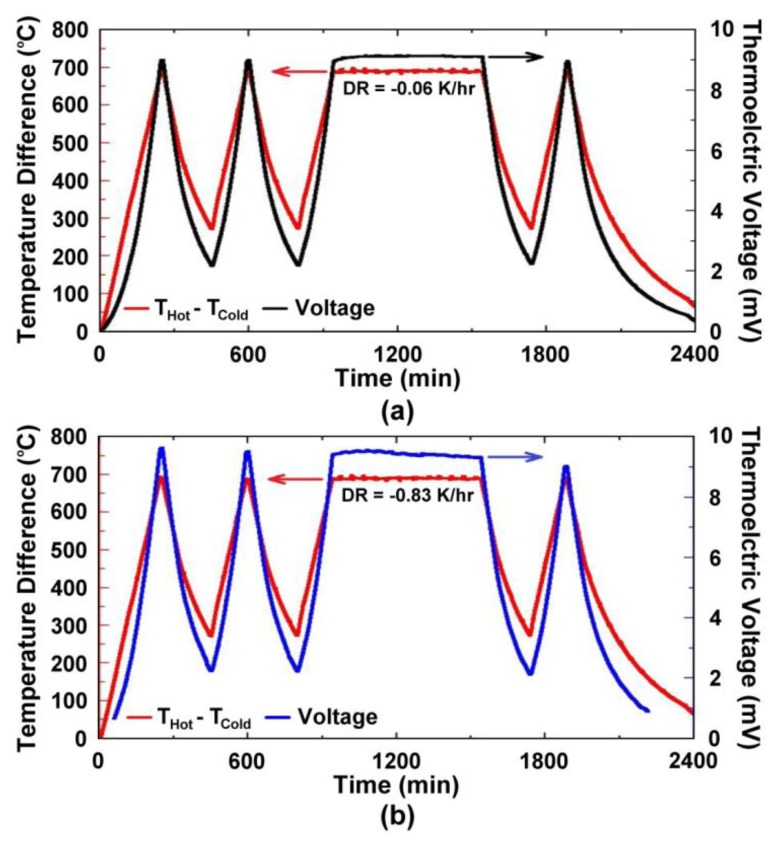
Thermoelectric output of two different platinum:palladium thermocouples on (**a**) alumina and (**b**) mullite. A peak hot junction temperature of 900 °C was used in each case.

**Figure 5.**
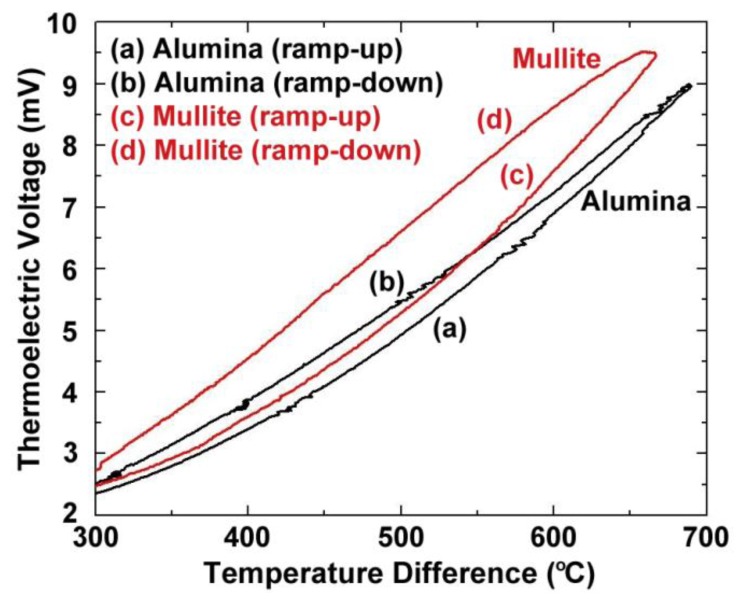
Hysteresis upon heating/cooling platinum:palladium thermocouples on alumina and mullite. Corresponds to second cycle of thermoelectric data in Figure 4.

Figure 4 shows the thermoelectric response of several platinum:palladium thin film thermocouples where a temperature difference as large as 750 °C was established along the length of the ceramic substrates and a peak temperature of 900 °C was achieved. The maximum thermoelectric voltage at peak temperature was 9.00 mV on alumina substrates and 9.52 mV on mullite substrates. This thermoelectric response was comparable to a conventional type-S wire thermocouple [19]. The Pt:Pd thin film thermocouples were more stable than both type-K and type-S over many thermal cycles with only slight decreases in peak thermoelectric voltage with each ramp in temperature due to microstructural changes such as grain growth, pore growth, and dewetting of the film, which was later confirmed by SEM imaging [16]. The hysteresis of these thin film thermocouples was smaller than that seen for type-K and type-S, where for the type-K it was much harder to measure due to significant instability in the thermoelectric response. Figure 5 shows the hysteresis for the Pt:Pd thin film thermocouples upon heating and cooling to 900 °C. In an ideal case, the thermoelectric voltage would be identical on the ramp up and ramp down. However, these thin film thermocouples had low hysteresis especially when compared to the metallic thin film thermocouples they were compared against. Here, the thin film thermocouples on mullite exhibited greater hysteresis than those formed on alumina.

**Figure 6.**
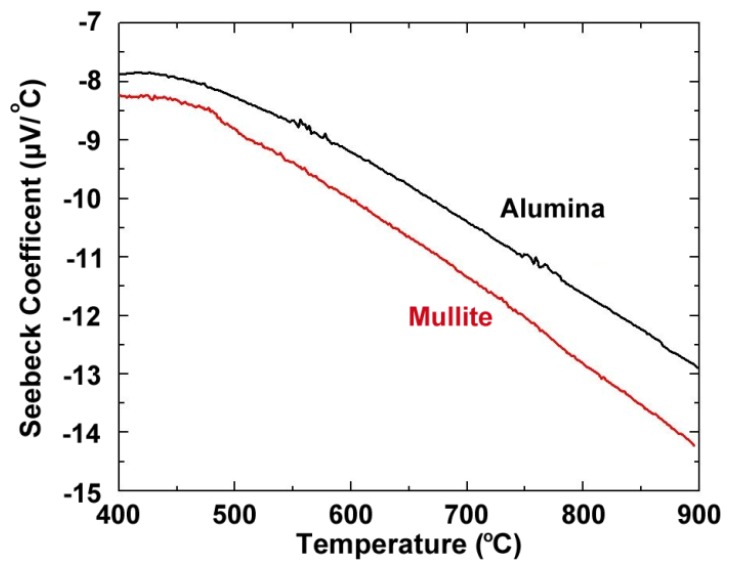
Seebeck coefficient of platinum:palladium thermocouples on alumina and mullite as a function of temperature and substrate.

**Figure 7.**
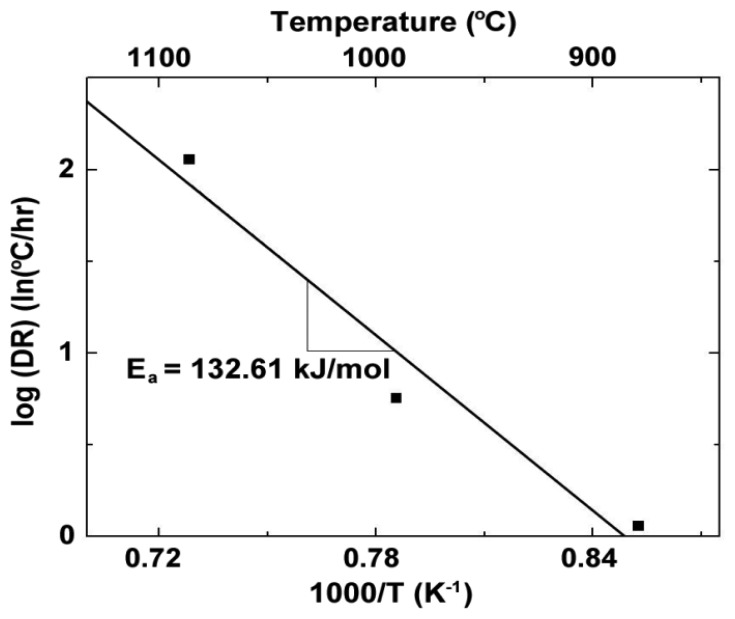
Drift rates of platinum:palladium thermocouples as a function of temperature, showing the activation energy calculated from the Arrhenius dependance.

The Seebeck coefficient of metal thermocouples is described by Equation (4):

(4) $$S(N_{D})=-{\left(\frac{\pi }{3N_{D}}\right)}^{⅔}\left(\frac{8k^{2}m^{*}T}{3e\hbar^{2}}\right)\left(A+\frac{3}{2}\right)$$

where *N_D_* is the carrier concentration, *k* is the Boltzmann constant, m* is the effective electron mass, *T* is the absolute temperature, *e* is the electron charge, and *A* is a transport constant [5]. There is no appreciable change in the carrier concentration of the platinum and palladium thin films over a large temperature range, so it was expected that the Seebeck coefficient increased in a linear fashion over most of the temperature range investigated. This was confirmed and shown in Figure 6. Thermocouple drift was defined according to Equation (5):

(5) $$DR(T)=\frac{\Delta V(T)}{V{\left(T\right)}_{\text{ref}}}\cdot \frac{T}{\Delta t}$$

where *DR*(*T*) is the drift rate given in °C/h, Δ*V*(*T*) is the change in voltage at constant temperature, *V*(*T*)*_ref_* is the initial voltage, *T* is the temperature, and *Δt* is the elapsed time at temperature. The drift rates of Pt:Pd thin film thermocouples were less than 1 °C/h in magnitude at temperatures up to 1,000 °C. However, microstructural changes in the films during thermal cycling were responsible for the observed drift and this was determined by modeling the drift rates as an Arrhenius dependence on temperature, which was modeled by Equation (6):

(6) $$DR(T)=A\cdot \exp\left(-\frac{E_{a}}{RT}\right)$$

where *A* is a constant, *E_a_* is the activation energy, *R* is the gas constant (8.314 J/mol·K), and *T* is the absolute temperature. Figure 7 shows the Arrhenius temperature dependence on drift rate for thin film Pt:Pd thermocouples deposited on alumina. The activation energy associated with the drift rates of the Pt:Pd thin film thermocouples was 132.61 kJ/mol, which falls between the activation energies for surface diffusion and volumetric diffusion of platinum and palladium. Therefore, a combination of surface and bulk microstructural changes caused the observed drift [20]. Decreasing the residual stress in the film from fabrication and reducing the surface free energy were the driving forces for these microstructural changes to occur. Directly observable evidence of these processes was seen in the constrained grain growth and dewetting of the thin films after thermal cycling.

#### 3.1.3. Ceramic Oxide and Oxynitride Thin Film Thermocouples

**Figure 8.**
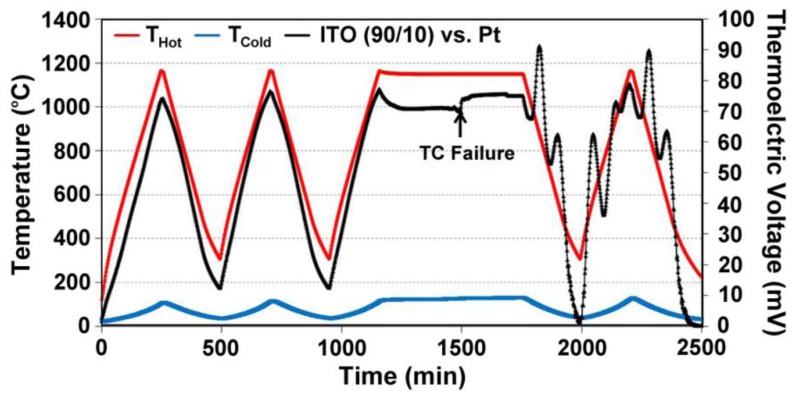
Thermoelectric output of a Pt:ITO 90/10 thermocouple tested after a 5 h, 500 °C nitrogen anneal but no air anneal. Note the progressive instability in the voltage signal with time until incoherence and noise in the signal are dominant. The film thickness was 6 μm. The point of device failure is indicated.

**Figure 9.**
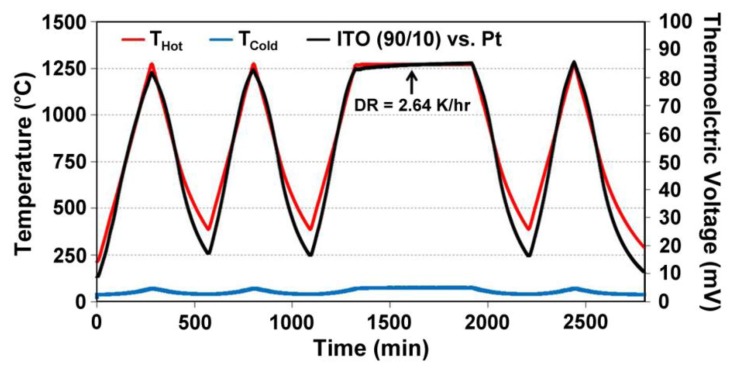
Thermoelectric output of a Pt:ITO 90/10 thermocouple tested after a 5 h, 500 °C nitrogen anneal and 1 h, 1,000 °C air anneal. Note the improvement in signal drift and device durability over the same testing period. The film thickness was 6 μm. A drift rate of 2.64 °C/h was observed.

Unbalanced compensation of oxygen vacancies in In_2_O_3_-based thin film thermocouples leads to inhomogeneities between the cold and hot junction microstructures, which lead to drift and eventually device failure during high temperature cycling. Data from a Pt:ITO 90/10 thin film thermocouple, which was not air-annealed prior to testing is shown in Figure 8. ITO was tested against a platinum reference leg to measure its true response. During the soak period there is noticeable drift to a point where the signals become unstable at a constant temperature difference and eventually become noisy and incoherent. At this point, there was enough difference in the microstructures and carrier concentration in the semiconductors used to form the hot and cold junction to cause the device to perform unstably. Therefore, all ceramic thin film thermocouples in this study were air annealed prior to testing to avoid premature device failure. Figure 9 shows an equivalent Pt:ITO thin film thermocouple which was annealed in air prior to testing. Note the direct improvement in signal drift and device durability over the same testing period.

**Figure 10.**
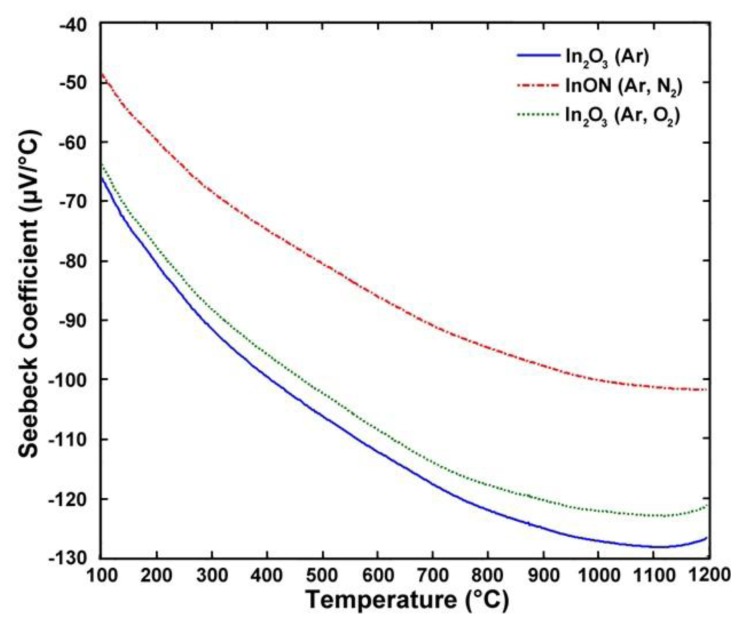
Seebeck coefficient of In_2_O_3_ films prepared in Ar, Ar/N_2_, and Ar/O_2_ plasmas. Note the lower Seebeck coefficient of the nitrided film relative to the films processed in argon and oxygen ambients. This was due to nitrogen acting as a valance band acceptor and lowering the carrier concentration in these films.

**Figure 11.**
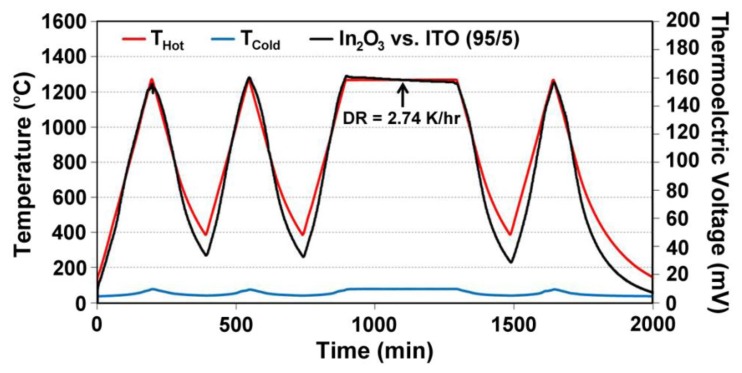
Thermoelectric output of a In_2_O_3_:ITO 95/5 thermocouple tested after a 5 h, 500 °C nitrogen anneal and 2 h, 1,000 °C air anneal. Note the stability of this all-ceramic thin film thermocouple over the testing period. A drift rate of 2.74 °C/h was observed.

**Figure 12.**
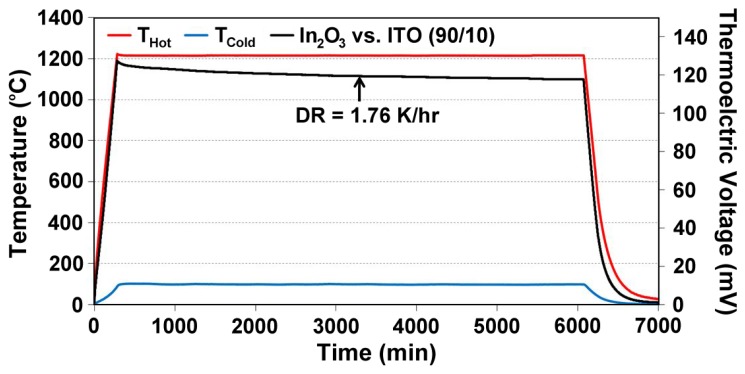
Thermoelectric output of a In_2_O_3_:ITO 90/10 thermocouple tested over a 100 h period after a 5 h, 500 °C nitrogen anneal and 2 h, 1,000 °C air anneal. The long term stability of this thin film thermocouple was measured in terms of an observed 1.76 °C/h.

**Figure 13.**
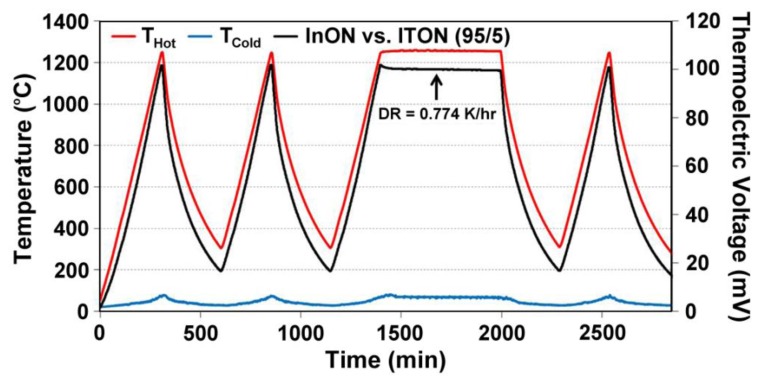
Thermoelectric output of a InON:ITON 95/5 thermocouple tested after a 5 h, 500 °C nitrogen anneal and 2 h, 1,000 °C air anneal. Note the reduced drift rate of this nitrided all-ceramic thin film thermocouple over its oxide counterpart (Figure 11). An order of magnitude improvement in the drift rate was observed at 0.774 °C/h.

**Figure 14.**
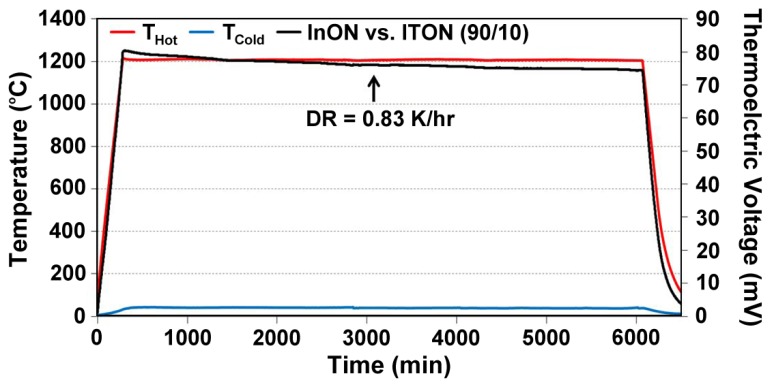
Thermoelectric output of a InON:ITON 90-10 thermocouple tested over a 100 h period after a 5 h, 500 °C nitrogen anneal and 2 h, 1,000 °C air anneal. The long term stability of this thin film thermocouple was measured in terms of an observed 0.83 °C/h drift rate, which is an order of magnitude improvement over its oxide counterpart (Figure 12).

**Figure 15.**
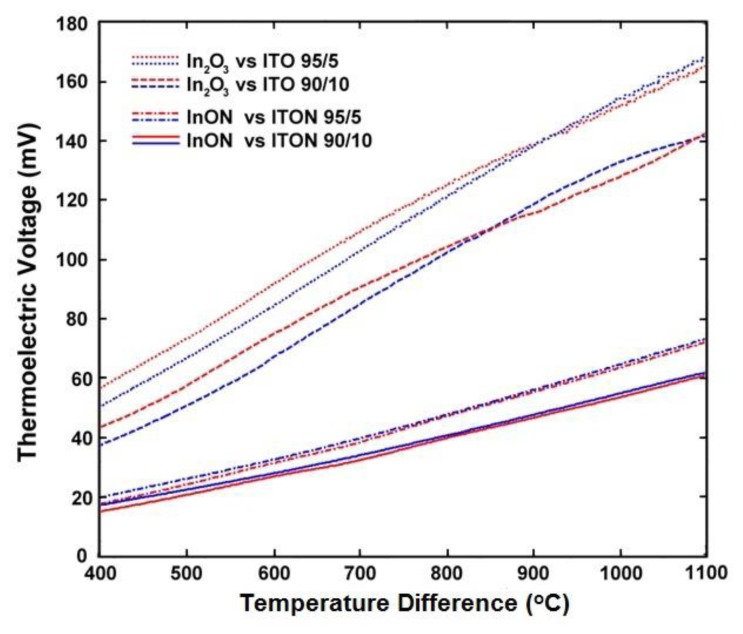
Thermoelectric output and hysteresis (heating: red, cooling: blue) of all-ceramic thin film thermocouples based on In_2_O_3_ and ITO.

The effects of reactive sputtering on the thermoelectric output of In_2_O_3_ was investigated and the Seebeck coefficient of each is shown in Figure 10. These thermocouples were tested relative to platinum reference electrodes to measure the true output and it was found that the films prepared in pure argon and in oxygen rich plasmas had larger magnitude Seebeck coefficients than those deposited in nitrogen-rich plasmas. For non-degenerate semiconductors, such as In_2_O_3_, a decrease in the carrier concentration increases the magnitude of the Seebeck coefficient. The Seebeck coefficient of non-degenerate semiconductors is described by Equation (7):

(7) $$S(N_{D})=-\frac{Ak}{e}-\frac{k}{e}\ln\left(\frac{{\left({2\pi \text{m}}_{\text{e}}^{*}\text{kT}\right)}^{2/3}}{\hbar^{3}{\text{N}}_{\text{D}}}\right)$$

where *S* is Seebeck, *k* is the Boltzmann constant, *e* is the electron charge, m_e_* is the effective mass, *ħ* is Planck's constant, and *A* is a transport constant (typically 0 ≤ A ≤ 4) [21]. It is expected that the oxynitride films will have a reduced Seebeck coefficient since nitrogen acts as a valence band acceptor in semiconducting oxides, reducing the magnitude of the thermoelectric output of these materials. The Seebeck coefficient of ITO is given by Equation (4), the same expression used for metals, which is valid because of the free electron-like behavior in degenerate semiconductors. Incorporation of nitrogen into ITO has been shown to reduce the carrier concentration as well as the Fermi level due to an increase in activation energy which increases the Seebeck coefficient of ITO [22]. Figures 11 and 12 show the thermoelectric output of an In_2_O_3_:ITO thin film thermocouple over many cycles and a 100 h heat soak test, respectively. The respective oxynitride equivalents are shown in Figures 13 and 14. It was observed that drift rates were reduced by an order of magnitude for the oxynitrides relative to the oxides but at the compromise of lower overall thermoelectric output. The Seebeck coefficients also decreased as a result, which was expected based on Equations (4) and (7). Additionally, the hysteresis in the thermoelectric output during thermal cycling was reduced by nitrogen processing, which is shown in Figure 15. Minimal hysteresis reduces the uncertainty in the voltage signals from the thin film thermocouple and makes for a more reliable sensor in harsh environments.

Parameters for equations relating the temperature difference to the thermoelectric voltage of the thin film thermocouples tested in this study, as well as their drift rates, are presented in Table 2. For both metallic and ceramic thin film thermocouples, a cubic polynomial was chosen as the model of best fit based on the hysteresis curves in Figures 5 and 15. Additionally, Table 3 presents the drift rates for the thin film thermocouples tested. Note the order of magnitude reduction in drift rates for Pt:Pd *versus* type-S and InON:ITON *versus* In_2_O_3_:ITO thin film thermocouples. For comparison, a 0.5 mm diameter type K wire thermocouple had a drift rate of 0.18 K/h at a hot junction temperature of 1,100 °C [15]. While the oxynitride ceramic thermocouples were not able to achieve drift rates lower than their metal wire counterparts, the stability was reduced to the same order of magnitude while providing the performance and versatility of thin film instrumentation.

### 3.2. Electrical Resistivity of Ceramic Thin Film Thermocouples

**Figure 16.**
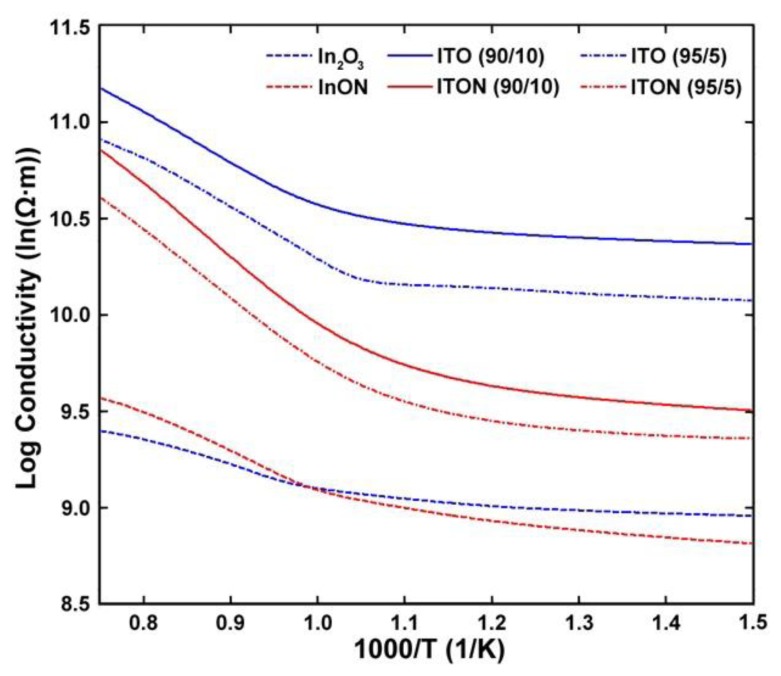
Electrical resistivity of In_2_O_3_ and ITO films prepared in Ar and Ar/N_2_ plasmas.

Arrhenius plots showing the electrical conductivity and activation energy of both argon and nitrogen processed In_2_O_3_ and ITO films are displayed in Figure 16. The activation energies corresponding to temperatures above and below the observed transition temperature are listed in Table 4. The oxynitride thin films had anywhere from 40–60% higher activation energies than their oxide counterparts. This indicates that nitrogen was present in the bulk film because it acts as a valence band acceptor in oxide semiconductors which results in a reduction of the Fermi energy, explaining the shift in activation energy. In all cases, the oxynitride thin films were more resistive at room temperature than the oxide thin films.

### 3.3. Chemical Analysis, Crystallography and Microstructure

#### 3.3.1. Platinum:Palladium Thin Film Thermocouples

AES was used to determine the chemical composition of the platinum and palladium thin films. Palladium contained no oxidation and on platinum it was limited to less than 1 nm on the surface. Oxygen was detected at small atomic percentages but was sourced from exposed ceramic substrate where the thin film had dewetted as a result of thermal cycling. Oxygen solubility in platinum and palladium is very low (0.035 atomic percent in palladium at 4.0 × 10^4^ Pa oxygen partial pressure and 850–900 °C) so it was not expected that the oxygen detected was from platinum and palladium oxides [23]. Above 500 °C, instead of forming a surface oxide, platinum evaporates as platinum metal or PtO_2_ and the extent to which this occurs is proportional to temperature and oxygen partial pressure [24,25]. Often, PtO_2_ decomposes and redeposits platinum metal on the surface of the film so that the overall material loss is minimal when compared to other noble metals [24]. Therefore, the oxygen detected at thicknesses less than 1 nm on the surface of platinum were likely from oxides which formed during cooling from elevated temperature and formed below 500 °C. No oxidation was found on the surface of the palladium thin films which is expected because it is much more likely that palladium will evaporate as a metal or as one of its volatile oxide, such as PdO, at high temperature [24]. Surface oxides formed on palladium decompose at temperature greater than 850 °C, and above 1,000 °C, the vapor pressure of palladium metal exceeds PdO at atmospheric pressure, so material loss from the thin film is likely palladium metal [24,25]. Depth profiling via AES revealed that platinum and palladium interdiffused at the metallurgical junction to form a complete solid solution of 90/10 atomic percent Pt/Pd on alumina and 78/22 atomic percent Pt/Pd on mullite, which is expected based on the phase diagram for this system [26].

**Figure 17.**
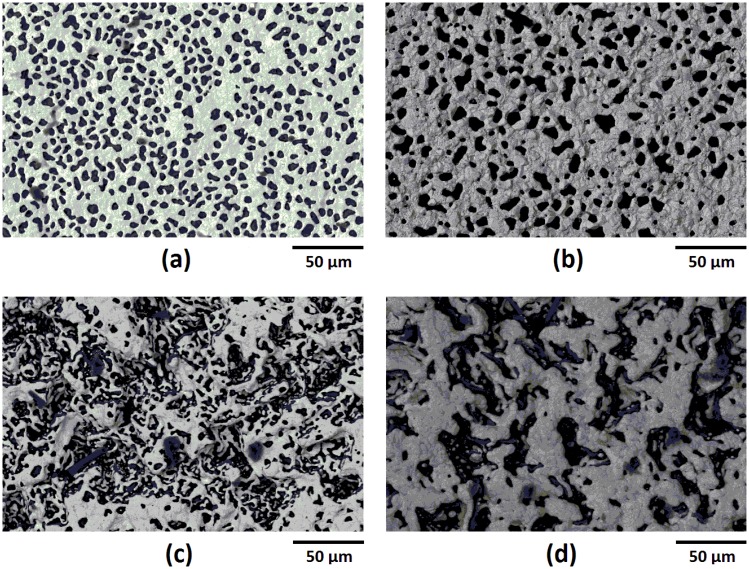
SEM micrographs using backscatter electron imaging (BSEI) of various thin film thermocouple legs after high temperature cycling: (**a**) platinum on alumina; (**b**) palladium on alumina; (**c**) platinum on mullite; and (**d**) palladium on mullite. Each film exhibited dewetting with distinctly different microstructures due to long term high temperature exposure.

**Figure 18.**
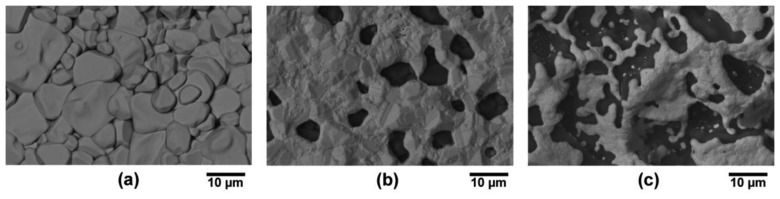
(**a**) SEM micrographs (BSEI) of an as deposited platinum film on alumina, the faceted striations in the thermocouple legs after high temperature cycling: (**b**) palladium on alumina; (**c**) platinum on mullite.

The microstructures of platinum and palladium thin films on both alumina and mullite were found to exhibit dewetting and faceting after thermal cycling. This was evident due to exposed substrate through area in which the film once covered as well as recrystallization from the faceting of pore edges at grain boundaries in particular. Palladium films contained larger pore sizes and grains than platinum which is due to its lower melting temperature and four order of magnitude higher surface recession rate than platinum [24]. The micrographs of each films are presented in Figure 17 where it is seen that each film exhibited dewetting, the palladium to a larger extent than platinum. All films remained contiguous after thermal cycling despite dewetting. The topography of the films on the mullite substrate appears much different than those on alumina because of the difference in surface roughness of each substrate. The mullite, being rougher than the alumina, induced more residual stress and surface energy in the thin films which increased the driving force for dewetting. The microstructures appear similar to those formed via the vapor phase transport mechanism in which continuous faceting of the metal film resulted from the dewetting process [2]. Faceted striations in the thin films, which is indicative of the vapor phase transport, were imaged at higher magnifications in Figure 18. Dewetting occurred at the metallurgical junction but to a lesser extent than the individual platinum and palladium legs since the junction was thicker and faceted striations were also observed. Delamination at the overlap of the metallurgical junction with the palladium leg was a result of the dewetting process in which the palladium leg had receded from the junction. While the thermocouple still functioned properly despite the recession of the film, delamination and dewetting ultimately lead to device failure where, in the limit, the platinum and palladium films would debond completely, most likely at the junction/thermocouple leg interface.

#### 3.3.2. Ceramic Oxide and Oxynitride Thin Film Thermocouples

**Figure 19.**
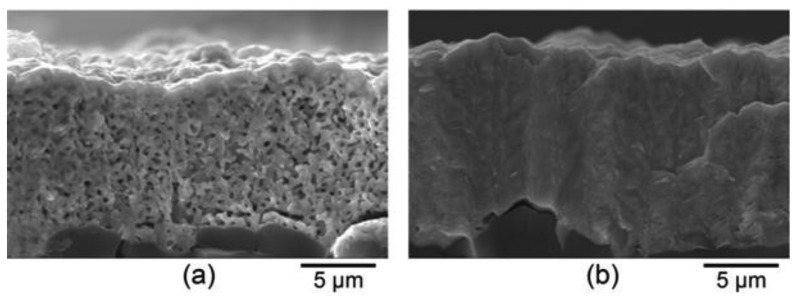
SEM micrographs of a (**a**) InON film fracture surface and a (**b**) In_2_O_3_ film fracture surface on compact alumina after annealing in air at 1,250 °C for ten hours. A densified layer is formed on the surface of the InON film and significant porosity can be observed in the bulk compared to the dense In_2_O_3_ film.

**Figure 20.**
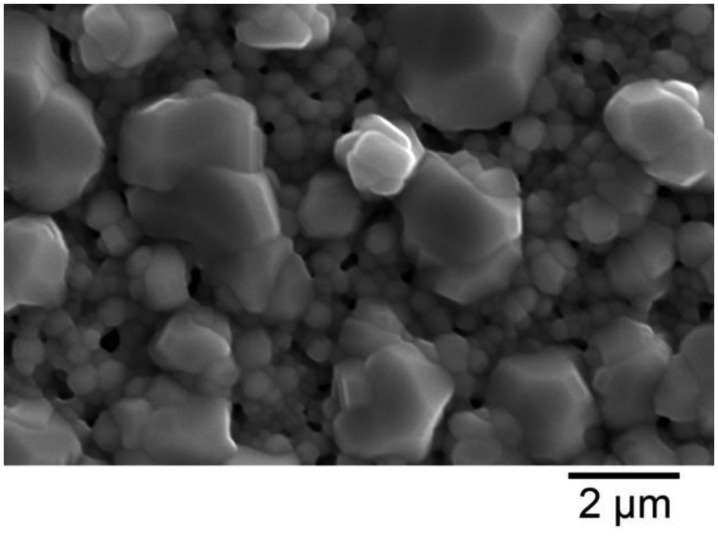
SEM micrograph of InON film prepared on c-axis sapphire after annealing in air at 1,250 °C for ten hours. Note the faceted particles and porosity. Less porosity is seen here relative to the fracture surface of the same film (Figure 19b).

**Figure 21.**
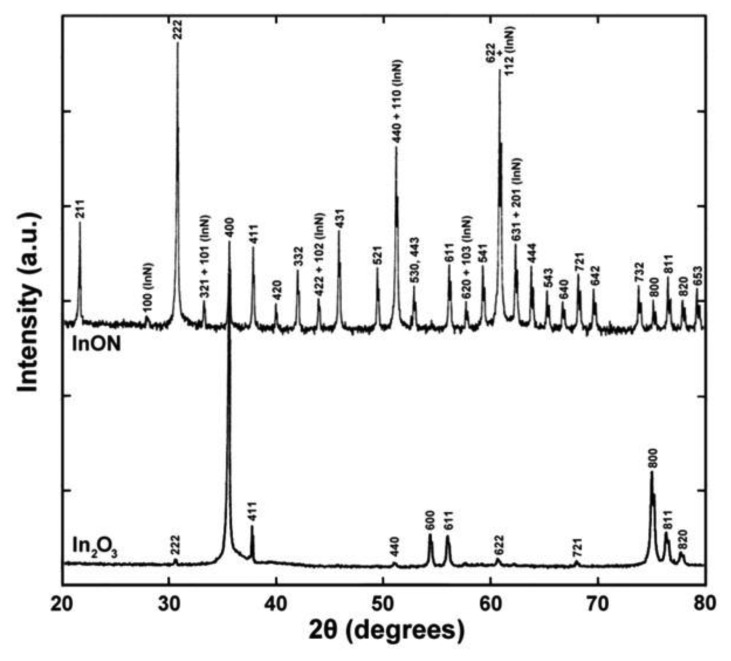
X-ray diffraction patterns of In_2_O_3_ and InON films after annealing in air at 1,250 °C for ten hours. Peaks corresponding to In_2_O_3_ and InN were present in the InON film indicating that metastable nitrogen was retained in the film as part of an indium nitride phase.

The microstructure of thermally cycled InON and In_2_O_3_ films are shown in Figure 19 where the InON film contained submicrometer pores in cross-section which were capped with a densified oxynitride layer (Figure 19a). A dissimilar microstructure was seen for the densified Ar processed film (Figure 19b) which contained very little porosity and no densified layer. The appearance of faceted particles over a network of submicrometer grains (Figure 20) was only observed for InON films but not for any tin doped films (ITON) or In_2_O_3_. This is believed to be the result of a vaporization-condensation mechanism where InON growth was driven by the appearance of unstable phases of indium oxides at temperatures greater than 1,200 °C (In_2_O_3_ → In_2_O_(g)_ + O_2_ and In_2_O_3_ → 2InO_(g)_ + 1/2O_2_) [27] and oxygen-partial pressures [28]. Oxide and oxynitride films were deposited onto sapphire substrates and their x-ray diffraction patterns are shown in Figure 21. The In_2_O_3_ films were oriented based on a well defined (400) peak and the films processed in nitrogen contained peaks corresponding to both In_2_O_3_ and InN phases. A slight increase in the lattice spacing was observed in the InON films relative to the In_2_O_3_ films with an increase from 2.530 Å to 2.546 Å for the (400) peak. This has been found previously in ITON and other oxynitride materials and is an indication that nitrogen is retained in the film and the densified surface layer [18,29].

**Figure 22.**
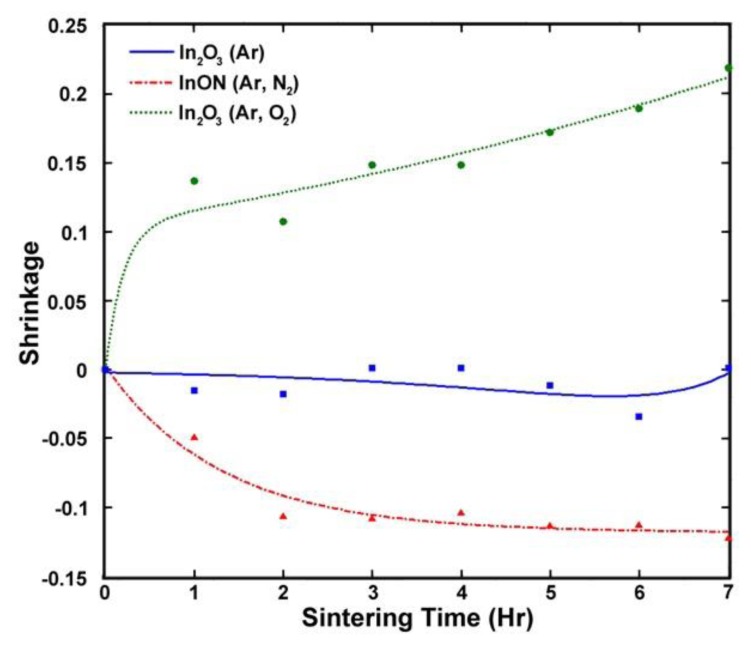
Isothermal constrained shrinkage ((V_0_−V)/V_0_) of In_2_O_3_ films prepared in Ar (blue square), Ar/O_2_ (green circle) and Ar/N_2_ (red triangle) plasmas at 1,300 °C as a function of time.

Kim *et al.* found that rapid densification of film surface grains constrains sintering in the bulk of the film, which suggests that nitrogen incorporated into the oxynitride film during deposition was metastably retained in pores and at grain boundaries due to the formation of the densified surface layer [30]. When the oxynitride films were thermally cycled, the slower sintering kinetics of nitrides relative to oxides resulted in pore growth and expansion in the bulk film as well as inhibited oxygen diffusion due to the densified surface layer. This phenomenon has been previously discovered in sintering of submicron silicon powder, MgO, ZnO, and SnO_2_ [31,32] and constrained volume shrinkage ([*V_0_*–*V*]/*V_0_*) due to this phenomenon has previously been investigated as a function of time and temperature [30]. Figure 22 shows the isothermal constrained volume shrinkage of In_2_O_3_ and InON films as a function of time at 1,300 °C in air. In_2_O_3_ prepared in Ar/O_2_ plasma was found to densify during the first hour at temperature followed by sublimation, and no significant change in thickness was observed for the argon processed film. InON experienced a 12% increase in volume after 2 h at 1,300 °C, which is indicative of the observed pore growth [31].

## 4. Conclusions and Outlook

Thin film instrumentation based on metallic and ceramic constituents have been developed for use in gas turbine engine applications to replace type-S, type K and other metallic thin film thermocouples, which suffer from oxidation and degradation during thermal cycling. Pt:Pd thin film thermocouples were more stable than type-S and type-K at temperatures over 1,000 °C. Additionally, by incorporating excess nitrogen into the ceramic films via reactive sputtering indium oxynitride-based thin film thermocouples were fabricated and outperformed their oxide counterparts in terms of drift rate and hysteresis. It was confirmed that metastable nitrogen was retained in the films, and due to the slow sintering kinetics of nitrides, the grain boundaries were stabilized and the formation of a densified surface layer on the film was also observed, which inhibited oxygen diffusion during thermal cycling. While not all thin film thermocouples outperformed their wire counterparts, many of the thin film thermocouples exhibited drift rates on the same order as wire thermocouples operating in the same temperature range. The ceramic-based thin film thermocouples exhibited an order of magnitude improvement in thermoelectric power relative to metal-based thin film thermocouples. Dewetting and related microstructural changes in the platinum and palladium thin films could be further inhibited by depositing thicker films (about 5–10 μm). Overall, the metallic and ceramic thin film thermocouples investigated here showed considerable promise as temperature sensors for harsh environments, such as the gas turbine engine hot section, due to their remarkable stability relative to currently available commercial instrumentation.

## Figures and Tables

**Table 1.** Sputtering parameters used for the deposition of thin film bond pads and thermocouple legs.

**Sputtering** **Parameters**	**Target** **Diameter** **(cm)**	**Target** **Power** **(W)**	**Power** **Density** **(W/cm**^**2**^**)**	**Sputtering** **Gas Pressure** **(Pa)**	**Deposition** **Rate** **(μm/h)**	**Thermocouple** **Film Thickness** **(μm)**
**Pt**	10.16	200	3.88	1.20 Ar	0.6	1.5
**Pd**	12.70	200	2.49	1.20 Ar	1.0	2.0
**In**_**2**_**O**_**3**_	15.24	350	1.98	1.33 Ar	1.2	10–12
**InON**	15.24	350	1.98	1.07 Ar 0.27 N_2_	1.1	10–12
**ITO 90/10**	15.24	350	1.98	1.33 Ar	1.4	10–12
**ITO 95/5**	12.70	350	2.85		0.9		
**ITON 90/10**	15.25	350	1.98	1.07 Ar 0.27 N_2_	-	10–12
**ITON 95/5**	12.70	350	2.85			
**Alumel** **(type-K)**	15.25	200	1.10	1.20 Ar	-	-
**Chromel** **(type-K)**	15.25	200	1.10	1.20 Ar	-	-
**Pt:10%Rh** **(type-S)**	12.70	200	2.49	1.07 Pa	-	1

**Table 2.** Parameters for the relationship of thermoelectric voltage to temperature difference and drift rates of thin film thermocouples. For both metallic and ceramic thin film thermocouples, a cubic polynomial was chosen as the best empirical model for the output curves.

**Thin Film Thermocouple**	**V (ΔT)=A(ΔT)**^**3**^**+B(ΔT)**^**2**^**+C(ΔT)**		

	**A (mV/°C**^**3**^**)**	**B (mV/°C**^**2**^**)**	**C (mV/°C)**
In_2_O_3_*vs.* ITO (95/5)	−6.90 × 10^−7^	1.44 × 10^−4^	7.27 × 10^−2^
In_2_O_3_*vs.* ITO (90/10)	−7.78 × 10^−7^	1.41 × 10^−4^	6.33 × 10^−2^
InON *vs.* ITON (95/5)	−1.43 × 10^−8^	4.66 × 10^−5^	2.17 × 10^−2^
InON *vs.* ITON (90/10)	−1.61 × 10^−8^	5.47 × 10^−5^	2.54 × 10^−2^
Pt:Pd on alumina	2 × 10^−5^	5 × 10^−4^	8.44 × 10^−1^
Pt:Pd on mullite	1 × 10^−-5^	4.9 × 10^−3^	−2.7 × 10^−2^

**Table 3.** Drift rates of thin film thermocouples at maximum hot junction temperature during testing. Note the order of magnitude reduction in drift rates for the Pt:Pd thin film thermocouples *versus* type-S and oxynitride-based *versus* oxide-based.

**Thin Film Thermocouple**	**Drift Rate (°C/h)**	**Seebeck Coefficient (μV/°C)**
In_2_O_3_*vs.* ITO (95/5)	3.76 (@ 1200 °C)	−897.7 (@ 1200 °C)
In_2_O_3_*vs.* ITO (90/10)	20.4 (@ 1200 °C)	−1,065.4 (@ 1200 °C)
InON *vs.* ITON (95/5)	0.57 (@ 1200 °C)	68.4 (@ 1200 °C)
InON *vs.* ITON (90/10)	0.63 (@ 1200 °C)	81.4 (@ 1200°C)
Pt:Pd on alumina	−0.06 (@ 900 °C)	−12.9 (@ 900 °C)
Pt:Pd on mullite	−0.83 (@ 900 °C)	−14.3 (@ 900 °C)

**Table 4.** Activation energies associated with the electrical conductivities of various oxide and oxynitride films as a function of thermal cycling. The activation energies come from the slope of the log of electrical conductivity *versus* 1/T curve.

**Target Material**	**Oxide Film**		**Oxynitride Film**	
	T < 950 K	T > 950 K	T < 950 K	T > 950 K
In_2_O_3_	5.86 meV	115 meV	8.68 meV	169 meV
ITO 95/5	16.1 meV	205 meV	25.1 meV	313.8 meV
ITO 90/10	13.5 meV	212 meV	21.3 meV	286 meV
